# Identification of Metabolomic Biomarkers of Seed Vigor and Aging in Hybrid Rice

**DOI:** 10.1186/s12284-022-00552-w

**Published:** 2022-01-27

**Authors:** Bing-Xian Chen, Hua Fu, Jia-Dong Gao, Yi-Xin Zhang, Wen-Jie Huang, Zhong-Jian Chen, Qi-Zhang, Shi-Juan Yan, Jun Liu

**Affiliations:** 1 Guangdong Key Laboratory for Crop Germplasm Resources Preservation and Utilization, Agro-biological Gene Research Center, Guangdong Academy of Agricultural Sciences, Guangzhou, Guangdong China; 2 Rice Research Institute, Guangdong Academy of Agricultural Sciences, Guangzhou, Guangdong China; 3 College of Agronomy, Hunan Agricultural University, Changsha, Hunan China

**Keywords:** *Oryza sativa*, Seed vigor, Seed aging, Metabolomic biomarkers, Galactose, Gluconic acid

## Abstract

Seed deterioration during rice seed storage can lead to seed vigor loss, which adversely affects agricultural production, the long-term preservation of germplasm resources, and the conservation of species diversity. However, the mechanisms underlying seed vigor maintenance remain largely unknown. In this study, 16 hybrid rice combinations were created using four sterile lines and four restorer lines that have been widely planted in southern China. Following artificial aging and natural aging treatments, germination percentage and metabolomics analysis by gas chromatography–mass spectrometry was used to identify the metabolite markers that could accurately reflect the degree of aging of the hybrid rice seeds. Significant differences in the degree of seed deterioration were observed among the 16 hybrid rice combinations tested, with each hybrid combination having a different germination percentage after storage. The hybrid rice combination with the storage-resistant restorer line Guanghui122 exhibited the highest germination percentage under both natural and artificial storage. A total of 89 metabolic peaks and 56 metabolites were identified, most of which were related to primary metabolism. Interestingly, the content of galactose, gluconic acid, fructose and glycerol in the seeds increased significantly during the aging process. Absolute quantification indicated that galactose and gluconic acid were highly significantly negatively correlated with the germination percentage of the seeds under the different aging treatments. The galactose content was significantly positively correlated with gluconic acid content. Additionally, glycerol showed a significant negative correlation with the germination percentage in most hybrid combinations. Based on the metabolomics analysis, metabolite markers that could accurately reflect the aging degree of hybrid rice seeds were identified. Galactose and gluconic acid were highly significantly negatively correlated with the germination percentage of the seeds, which suggested that these metabolites could constitute potential metabolic markers of seed vigor and aging. These findings are of great significance for the rapid and accurate evaluation of seed aging degree, the determination of seed quality, and the development of molecular breeding approaches for high-vigor rice seeds.

**Supplementary Information:**

The online version contains supplementary material available at 10.1186/s12284-022-00552-w.

## Background

Rice (*Oryza sativa* L.) is one of the most important food crops for human beings, serving as a major staple for over half of the world's population. Rice production is crucial in ensuring China's food security. Seed vigor refers to the sum of the properties that determine seed performance, such as the performance under field and storage conditions (Hampton and Tekrony 1995). Generally, rice seed vigor tends to decline under the high temperature and high humidity conditions of South China (Fu 1985). The percentage of hybrid rice seeds that germinate can be reduced to less than 70% after one year of storage in the rice cultivation region of southern China, thus resulting in significant production losses. Seed vigor is also related to the long-term storage of germplasm resources and the preservation of species diversity. Therefore, improving seed storability by maintaining seed vigor during storage is important in rice production (Aibara et al. 1986).

Multiple “omics” studies have demonstrated that many physiological, cellular, biochemical, and metabolic alterations occur during seed storage, and a number of genes related to the storability of rice seeds have been identified, including aldehyde dehydrogenase 7 (*OsALDH7*) (Shin et al. 2009), three rice lipoxygenase (*LOX*) isozymes (Zhang et al. 2007), and rice protein repair enzyme L-isoaspartyl methyltransferase PIMT1 (Aibara et al. 1986). However, the mechanisms regulating the maintenance of vigor during seed storage are still largely unclear. Alterations in the expression of genes or proteins during seed storage influence the production and metabolism of small molecules (Suzuki and Matsukura 1997). Thus, metabolomics, as a comprehensive, unbiased, high-throughput analysis of complex metabolite mixtures in target organisms, has been applied in several seed studies (Hall et al. 2002).

Metabolites are essential for plant development, providing energy for seeds, acting as defense signaling molecules, and serving as nutrients for humans and animals (Hu et al. 2015). It is generally believed that seed vigor relies on the accumulation of storage substances. As a seed matures, proteins, starches, and other substances accumulate, and the seed germination ability and vigor increase, reaching a peak at the physiological maturity stage (Bewley et al. 2012). At the later stages of seed development, stress resistance-related substances such as storage proteins, late embryo abundance (LEA) proteins, oligosaccharides, abscisic acid (ABA), and tocopherols accumulate, which contribute to seed vigor and enhance the adaptability of seeds to environmental stress (Sun et al. 2007). During storage, seeds undergo aging and deterioration, showing a decline in the total amount of storage substances and the degradation of storage proteins (Fu 1985; Wang et al. 2015).

Rice varieties vary in seed storability, which depends to some extent on the chemical composition of the mature seeds. Inositol galactosides and raffinose family oligosaccharides accumulate at the ripening stage in the seeds of some crops such as legumes, suggesting a role in the desiccation tolerance and longevity of seeds (Sengupta et al. 2015; Salvi et al. 2016).The galactinol content of mature dry seeds can be used as a biomarker for seed longevity in Brassicaceae species and tomato (de Souza Vidigal et al. 2016). The content of inositol galactosides is considered a marker of seed vigor in Arabidopsis, cabbage, and tomato (de Souza Vidigal et al. 2016).Through peroxidation, some polyunsaturated fatty acids generate end-products such as malondialdehyde, hydroxyl radicals, keto fatty acids, and other reactive oxygen molecules, which accumulate in the cells. If they reach a very high level, they cause damage to the cells by reacting with macromolecular substances (Pisoschi and Pop 2015). The peroxidation of polyunsaturated fatty acids is a major factor affecting seed storability (Xu et al. 2015). Alterations in the content of soluble sugars in seeds affect seed vigor (Bernal-Lugo and Leopold 1995). Raffinose, trehalose, and other oligosaccharides have been found to be related to seed desiccation tolerance (Bernal-Lugo and Leopold 1992; Nishizawa et al. 2008).

We previously compared the proteomic and metabolomic differences between control and hybrid combinations with poor storability and found that the seeds with poor storability exhibited differential increases in proteins and metabolite changes during natural aging. We also suggested that raffinose might be related to hybrid rice seed storability which partly reflects seed vigor (Bewley et al. 2012; Gao et al. 2016; Yan et al. 2018). Therefore, in the current study, we used more combinations with broader properties and additional aging treatments to identify reliable metabolic markers for assessing the seed vigor and aging of hybrid rice. In this study, four sterile lines and four restorer lines that have been widely planted in southern China were used to form 16 hybrid combinations. Through germination tests and metabolomics analysis using gas chromatography-tandem mass spectrometry (GC–MS) of the seeds after artificial and natural aging treatments, we found that the seeds of hybrid combinations derived from the restorer line ‘Guanghui122’ (which is tolerant to storage) were highly resistant to deterioration. Importantly, we also discovered that the contents of galactose and gluconic acid had a significant negative correlation with the seed germination percentage and thus could be used as metabolic markers for seed vigor and aging. The results of the study guide the molecular breeding of storage-tolerant rice and can inform the quality control of rice seeds during production.

## Results

### Seed Germination of Different Rice Hybrid Combinations Following Aging Treatment Differs Significantly

**Fig. 1:**
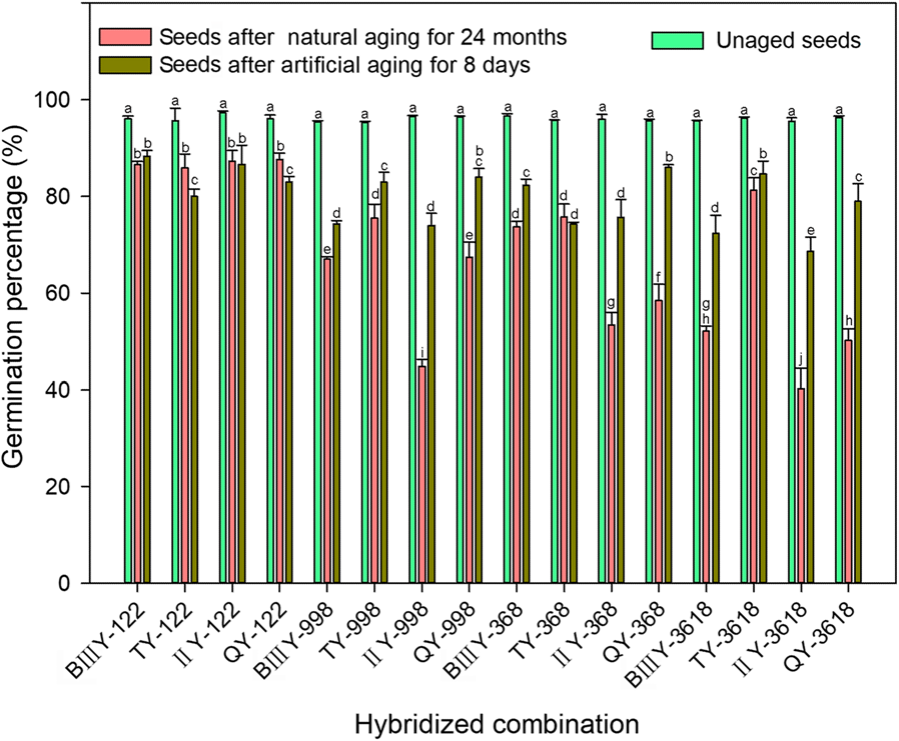
Changes in seed germination percentage of 16 hybrid rice combinations (BIIIY-122, TY-122, IIY-122, QY-122, BIIIY-998, TY-998, IIY-998, QY-998, BIIIY-368, TY-368, IIY-368, QY-368, BIIIY-3618, TY-3618, IIY-3618 and QY-3618) after 24 months of natural aging or 8 days of artificial aging. Data represent the mean ± SE of three biological replicates of 100 seeds each. Means denoted by the same letter did not significantly differ at p < 0.05 according to Fisher’s least significant difference test

Using 16 hybrid rice combinations as experimental materials, the harvested seeds were subjected to natural aging and artificial aging treatments, and the seed germination before and after aging was calculated (Fig. 1). There was no significant difference in the seed germination of the different combination before the treatment. However, after aging, the germination percentage of all combinations, regardless of the aging treatment, demonstrated a significant downward trend. There was also a significant difference in the degree of the decline in seed germination among the different combinations, indicating that the seed storage tolerance or seed vigor among the tested 16 hybrid rice combinations differed (Fig. 1).

After two years of natural aging, the seed germination of BIIIY-122, TY-122, IIY-122, QY-122, and TY-3618 was greater than 80%. Therefore, these five combinations were thus regarded as being resistant to storage under natural conditions, and those seeds were considered as high-vigor. By contrast, the germination percentages of IIY-998, IIY-368, QY-368, BIIIY-3618, IIY-3618 and QY-3618 were 45%, 53%, 58%, 52%, 40% and 50% respectively (Fig. 1). These six combinations were thus regarded as less resistant to natural aging, and were considered as low-vigor seeds in this study. The other combinations whose germination percentages between 60 and 80% were considered to be moderately vigorous.

The germination dynamics of the artificially aged seeds were similar to the naturally aged seeds. The germination percentage of the five combinations, BIIIY-122, TY-122, IIY-122, QY-122 and TY-3618, that were resistant to natural storage, was higher than 80% after artificial aging. For IIY-998, IIY-368, BIIIY-3618, IIY-3618 and QY-3618, which were not resistant to natural storage, the seed germination under artificial aging was also lower than 80% (Fig. 1).

### The Maintenance of Vigor During Seed Aging Varies According to the Parental Sterile Line and Restorer Line

**Fig. 2:**
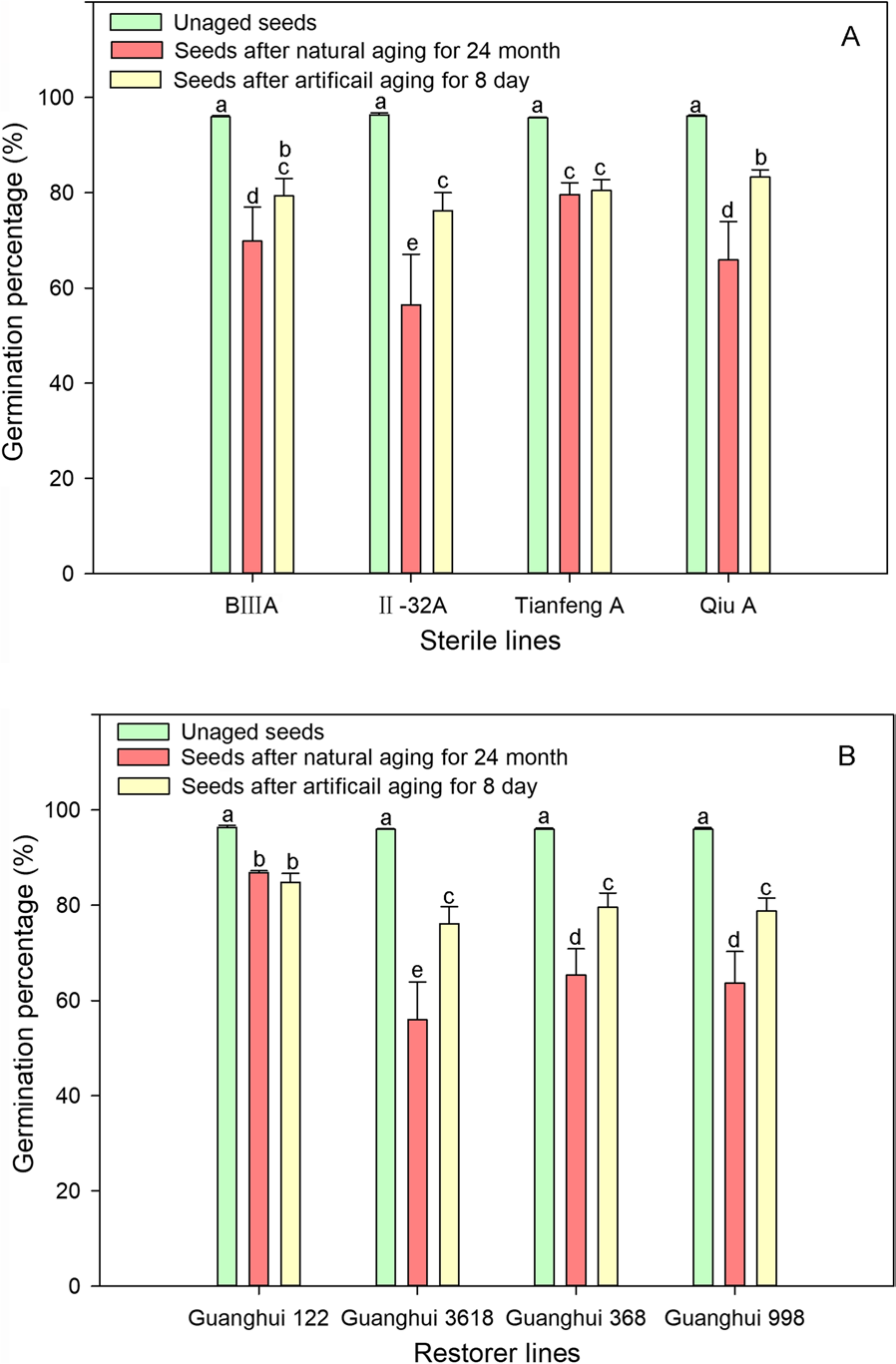
Changes in rice seed germination of 4 sterile lines (BIIIA, II-32A, Tianfeng A, Qiu A) (**A**) and 4 restorer lines (Guanghui122, Guanghui3618, Guanghui368, Guanghui998) (**B**) after 24 months of natural aging or 8 days of artificial aging. Data represent the mean ± SE of three biological replicates of 100 seeds each. Means denoted by the same letter did not significantly differ at p < 0.05 according to Fisher’s least significant difference test

Before storage or aging, no difference in the average germination percentage of the seeds among the sterile lines was detected. Conversely, the germination percentage differed significantly following natural aging or artificial aging, indicating that the sterile line affected the seed vigor retention ability or life span (Fig. 2A). The hybrid rice combinations of the II-32A sterile line had poor storage resistance except IIY-122, being lower than the average value of the other sterile lines after natural aging (Figs. 1, 2).

The overall trend of the restorer lines was similar to that of the sterile lines. There was no difference in the average germination percentage of the seeds of the four restorer lines before aging, but there was a significant difference after aging (Fig. 2B). The restorer line Guanghui 122 showed the highest germination percentage under both artificial and natural aging treatments, being significantly higher than that of the other three restorer lines, thus demonstrating good vigor maintenance capacity. Its germination after natural aging and artificial aging was 91% and 88%, respectively (Fig. 2B). The germination percentage of the hybrid combinations between Guanghui 122 and sterile lines BIIIY-122, TY-122, IIY-122 and QY-122 were 87%, 87%, 88%, and 87%, respectively (Fig. 1). However, the seeds of Guanghui 998 and Guanghui 368 showed only moderate storability, and their derived hybrid combinations presented weak storability (Figs. 2B, 1).

### Comparative Analysis of the Metabolomes of Hybrid Rice Seeds Before and After Natural Aging

To assess if the chemical composition of the hybrid rice seed was associated with its seed vigor or storability, we firstly compared the metabolome of 32 seed samples (16 before storage and 16 after 24-month storage) using a GC–MS-based metabolomics approach. A total of 89 metabolite peaks were detected, and 56 metabolites were identified based on our in-house database (Yan et al. 2018). Among them, 24 were identified as sugar-related compounds, 20 were amino acid-related compounds, 2 were free fatty acids, 6 were tricarboxylic acid cycle-related intermediates, and 4 were other compounds. Most of them were primary metabolites, which may reflect the physiological states of the seeds.

**Fig. 3:**
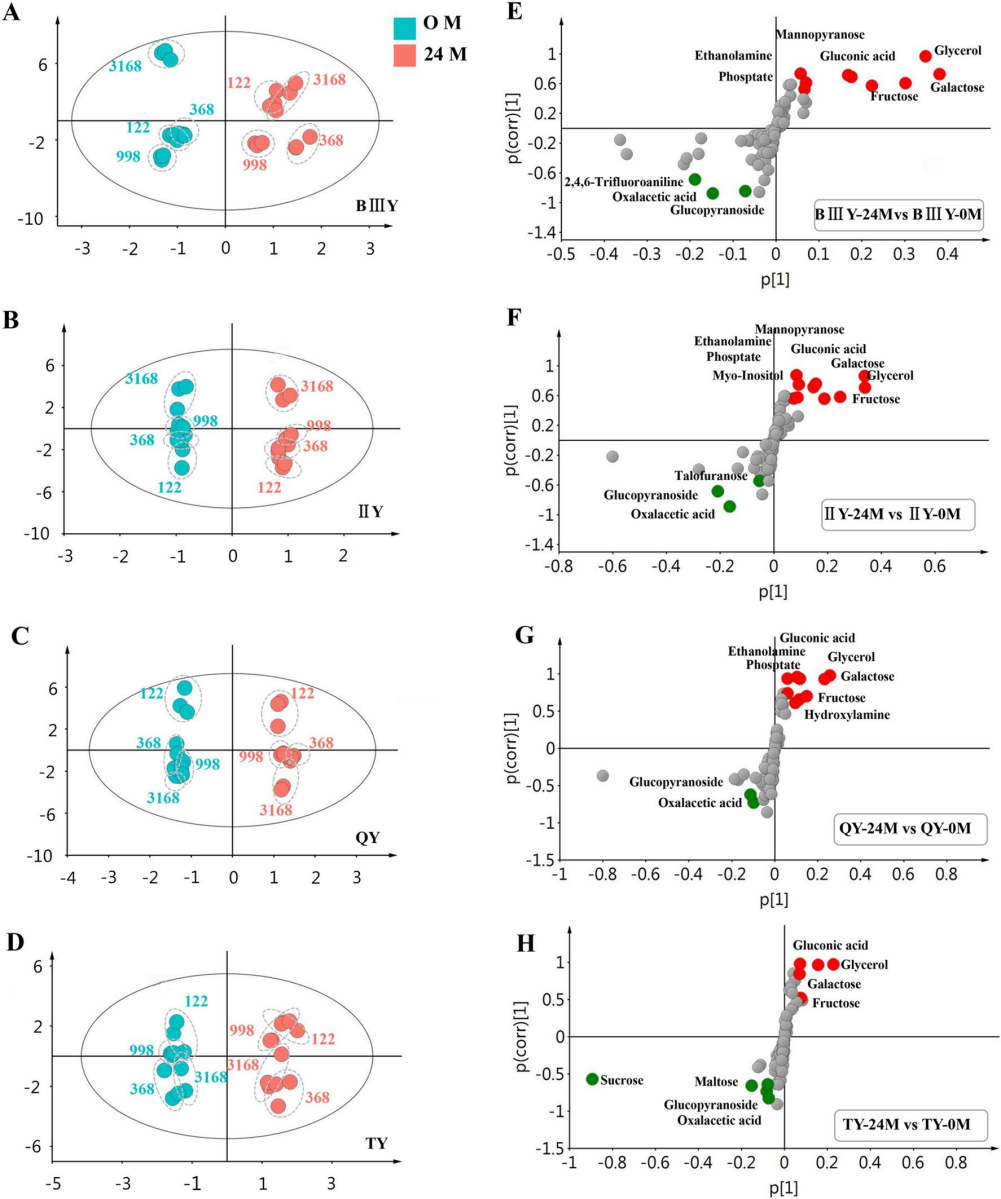
The score plots (**A**–**D**) and S-plots of the different metabolites (**E**–**H**) generated from pairwise OPLS-DAGC-MS data showing the distinct metabolomic changes among the BIIIY-122, BIIIY-998, BIIIY-368, BIIIY-3618 (**A**, **E**),IIY-122, IIY-998, IIY-368, IIY-3618 (**B**, **F**), QY-122, QY-998, QY-368, QY-3618 (**C**, **G**), and TY-122, TY-998, TY-368, TY-3618 (**D**, **H**). The significantly different metabolites with VIP > 0.5 and |p(corr)| > 0.5 have been labeled in the S-plot (**B**, **D**, **F**, **H**)

To generate an overview of the metabolic difference between all the samples, we performed multivariate statistical analyses of metabolomic data, including principle component analysis (PCA) and orthogonal partial least squares-discriminant analysis (OPLS-DA). First, the score plot derived from the PCA model indicated that the non-aged and aged seed samples were distributed in separate groups for all hybrid rice combinations based on the first two principal components, and there was no outlier sample (Additional file 1: Fig. S1), thus indicating distinct metabolic differences occurred during 24-month storage. Further, OPLS-DA model was also established to assess the metabolic patterns of these samples accurately, which can filter out irrelevant orthogonal signals. It is shown that the overall trends were similar to those observed in the PCA model, but more obvious in the score plot (Fig. 3A–D).

Finally, pairwise comparisons of the seed metabolome using orthogonal OPLS-DA were further performed to identify potential metabolite biomarkers that differed significantly in each comparison group. As shown in Fig. 3, 12 metabolites were significantly different between BIII A-0M and BIII A-24M (Fig. 3E; Additional file 4: Table S3); 13 were significantly different between II-32A-0M and II-32A-24M (Fig. 3F; Additional file 4: Table S4); 11 were significantly different between Qiu A-0M and Qiu A-24M (Fig. 3G; Additional file 4: Table S5); and 10 were significantly different between Tianfeng A-0M and Tianfeng A-24M (Fig. 3H; Additional file 4: Table S6). Among these metabolites, most of them were sugar metabolism-related metabolites, such as galactose, fructose, gluconic acid, glycerol (Additional file 4: Table S3–S6).

### Changes in Metabolites Related to Seed Aging During Storage

The differential metabolites were analyzed using univariate Student’s *t*-tests. Although we did not find any differently abundant amino acids and lipids related to seed vigor or seed aging, we did detect differential soluble sugars and their derivatives.

**Fig. 4:**
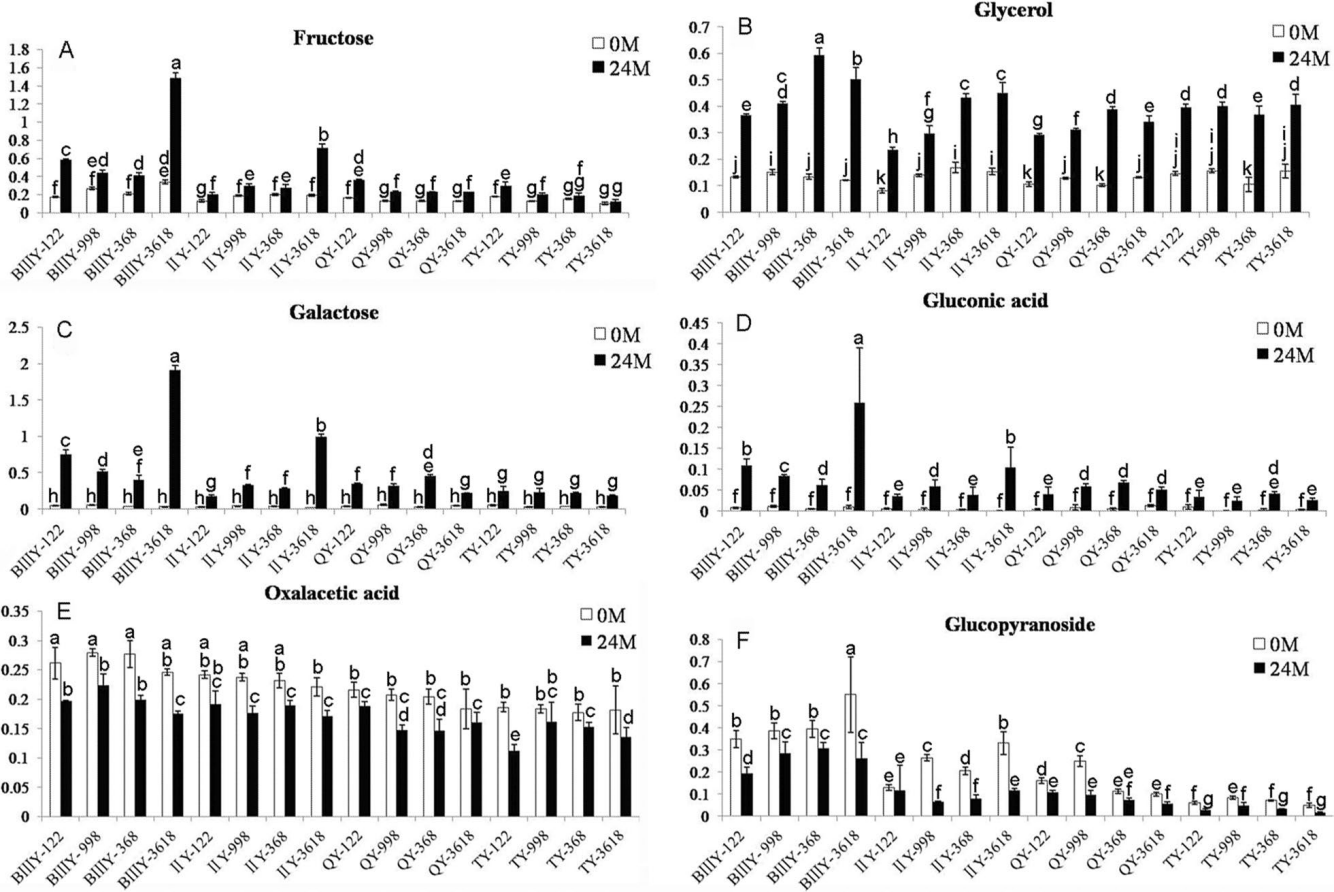
Changes in the relative contents of fructose (**A**), glycerol (**B**), galactose (**C**), gluconic acid (**D**), oxaloacetic acid (**E**) and glucopyranoside (**F**) in the seeds of 16 hybrid rice combinations by natural aging for 24 months and 0 mouths. Data represent the mean ± SE of six biological replicates of 100 mg seeds each. Means denoted by the same letter did not significantly differ at p < 0.05 according to Fisher’s least significant difference test

As shown in Fig. 4, the relative contents of galactose, fructose, gluconic acid, and glycerol in most of combinations increased significantly following 24 months of natural aging (Fig. 4A–D). Conversely, the levels of glucopyranoside and oxaloacetic acid in most of combinations decreased significantly after 24 months of natural aging in all samples (Fig. 4E, F).

Among these six sugar-related metabolites, both the levels of galactose and gluconic acid were significantly greater in the seeds after 24 months of natural storage, ranging between 4 and 50 times more for the former, and between 3 and 100 times more for the latter across the different combinations (Fig. 4C, D, Additional file 5: Table S7). Glycerol and fructose were two times higher in the seeds after 24 months of natural storage (Fig. 4A, B), while glucopyranoside and oxaloacetic acid decreased after the 24-month natural storage period (Fig. 4E, F, Additional file 5: Table S7). These results suggested that these six metabolites might constitute preliminary candidate markers for seed aging.

Among the other identified sugar-related metabolites, the levels of raffinose, galactinol, sucrose and myo-inositol remained relatively constant in all the 16 tested seeds during the 24-month natural storage period (Additional file 5: Table S7), which is similar to the findings in our previous study (Yan et al. 2018). Furthermore, we found that there was no significant correlation between the relative content of galactinol, sucrose, trehalose, and myo-inositol and seed germination (Additional file 6: Table S8). However, before storage, the correlation between raffinose content and seed germination under artificial aging (r = 0.5111) was significant (*p* < 0.05) (Additional file 6: Table S8). This confirmed our previous research that detected a positive correlation between raffinose content and seed vigor (Yan et al. 2018).

### Galactose, Gluconic Acid, Fructose, and Glycerol are Potential Markers of Seed Vigor and Aging

**Fig. 5:**
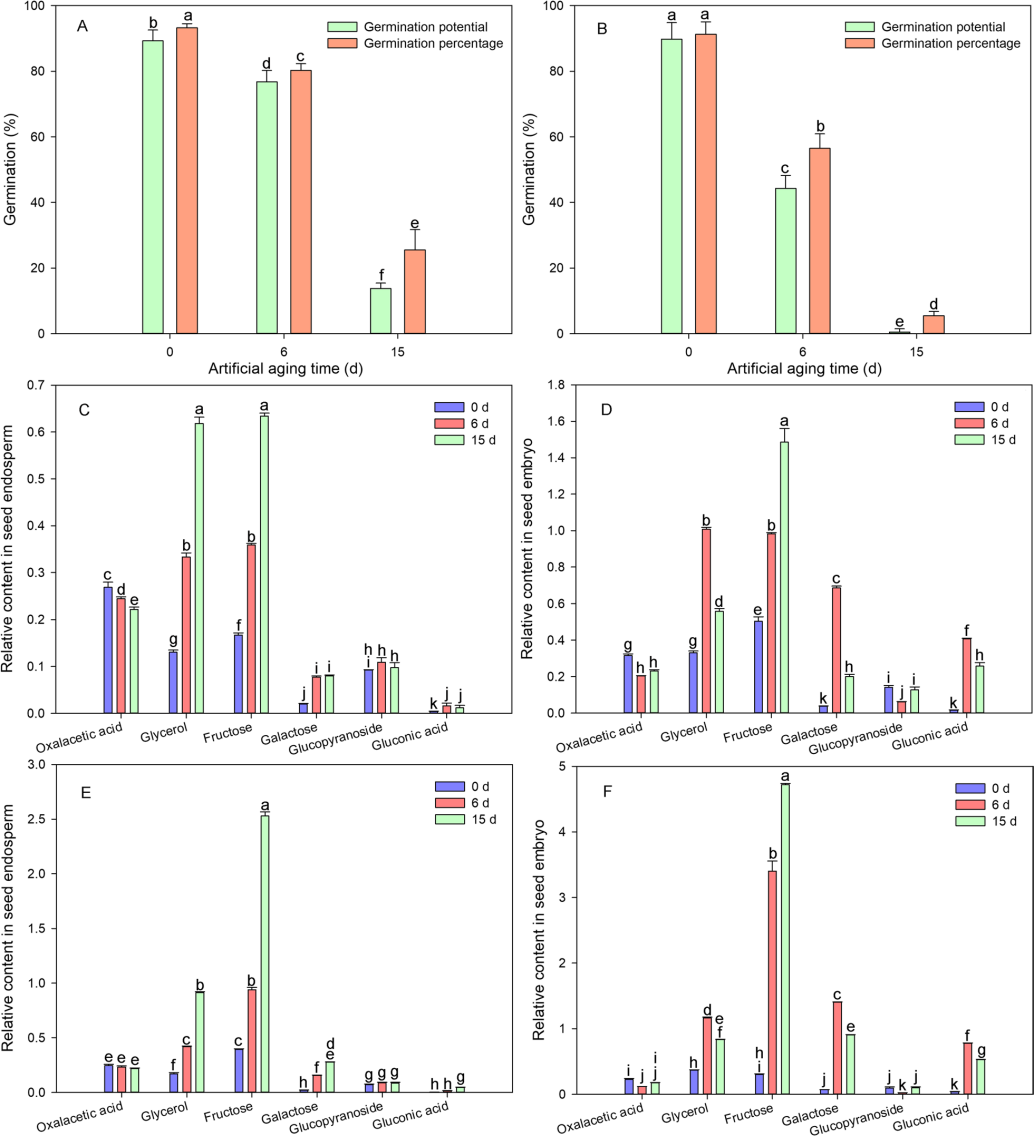
Changes in germination percentage and six metabolites in seeds of two hybrid rice combination after 0, 6 or 15 days of artificial aging. **A** Seed germination percentage of G8Y2156; **B** Seed germination percentage of G8Y169; **C** Relative content of six metabolites in seed endosperm of G8Y2156; **D** Relative content of six metabolites in seed embryo of G8Y2156; **E** Relative content of six metabolites in seed endosperm of G8Y169; **F** Relative content of six metabolites in seed embryo of G8Y169. Data represent the mean ± SE of six biological replicates of 100 mg seeds (endosperm or embryo) each. Means denoted by the same letter did not significantly differ at *p* < 0.05 according to Fisher’s least significant difference test

To further verify the potential role of these metabolites as markers of seed vigor and aging, we selected two combinations, namely G8Y2156 and G8Y169, and subjected them to artificial aging treatment for 6 d and 15 d, respectively, to obtain seeds with different degrees of aging. The candidate metabolites of the above-mentioned six types of seed aging degree markers were determined in the seed embryo and endosperm (Fig. 5).

The results showed that as seed germination decreased with the increase in aging treatment duration (Fig. 5A, B), the content of fructose, glycerol, galactose, and gluconic acid in the endosperm also increased (Fig. 5C, E). The change trend of these substances in the endosperm was consistent with the above results in the seeds under natural aging (Fig. 4). However, the metabolite changes in the embryo were not completely consistent with the above trend, and only the fructose content increased (Fig. 5D, F). These results also suggested that the contents of these sugars should be determined only in the endosperm, which can provide a semi non-destructive means of testing, with potential broad application prospects. The other two candidates, oxaloacetic acid and glucopyranoside, exhibited little relationship with seed aging. Therefore, combining the results of the changes in the six metabolites in the rice seeds treated by natural aging and artificial aging, we suggest that galactose, fructose, gluconic acid, and glycerol may serve as candidate markers of seed vigor.

### Regression Equations Based on Galactose and Gluconic Acid Content Predict the Seed Germination Percentage During Storage

We then used an absolute quantitative method to measure the galactose, gluconic acid, fructose, glycerol, oxaloacetic acid, glucopyranoside, and raffinose contents in the other rice combinations and restore lines (G8Y165, NXRZ, NYZ, G8B, HZ, RXZ, R534, M2YHZ, Y2Y1, JFY1002) stored at room temperature for different durations, comparing them against a galactinol control (a previous study showed that it is related to seed longevity or aging degree).The seed germination percentage of the 26 materials under natural storage for different durations ranged from 0 to 99% (Additional file 7: Table S9), indicating that the seeds differed in their aging degree and vigor. Among these metabolites, the correlation coefficient between galactose and gluconic acid and seed germination percentage was − 0.937 and − 0.935 respectively, indicating a highly significant negative correlation, and the correlation coefficient between the two metabolites was as high as 0.984 (Additional file 7: Table S9). In addition, glycerol contents was also negatively correlated with seed germination, with correlation coefficients between germination percentage and glycerol was − 0.723. Furthermore, the correlation coefficient between the sum of the absolute content of galactose, gluconic acid, and glycerol and seed germination reached − 0.940, indicating remarkably negative correlation (Additional file 7: Table S9).

The changes in oxaloacetic acid, glucopyranoside and fructose, were not significantly correlated with seed aging (Additional file 7: Table S9). Interestingly, although only the raffinose level was positively correlated with seed germination, this correlation coefficient was merely 0.230. Moreover, although the content of galactinol has been reported to be related to the storage capacity of seeds (de Souza Vidigal et al. 2016), it exhibited no significant correlation with the degree of natural aging in the rice seeds (Additional file 7: Table S9).

**Fig. 6:**
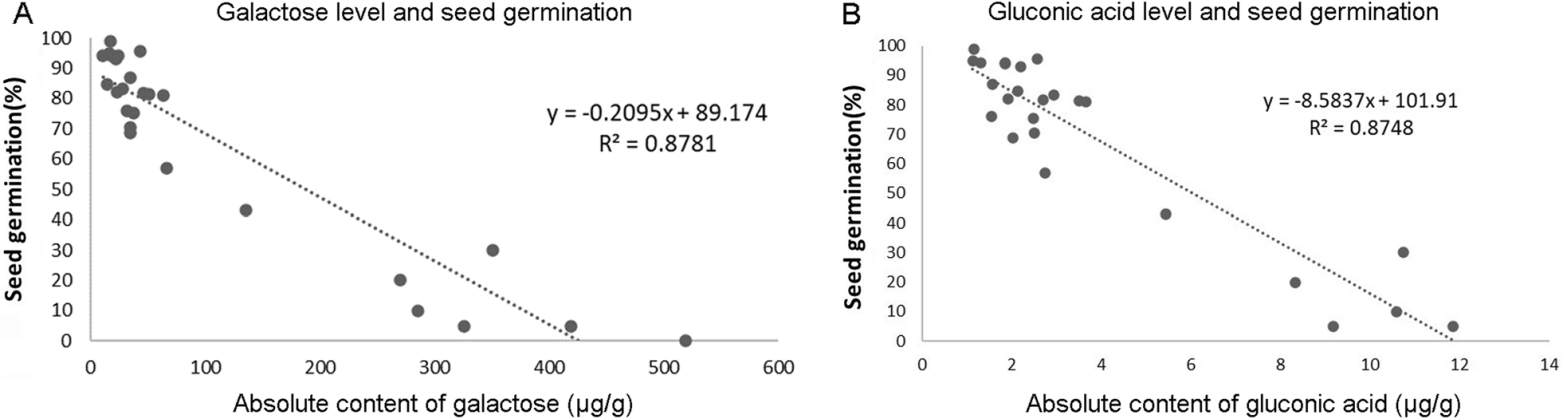
Regression equation between the absolute content of galactose (**A**) and gluconic acid (**B**) and different germination percentages of 26 rice materials with different seed vigor. Y represent germination percentage; R^2^ represent the determination coefficient

We then calculated the regression equation of galactose level, gluconic acid level and seed germination in order to predict the germination percentage of the seeds with various vigor during storage. The regression equation between the absolute content of galactose and the seed germination was: y = 89.174 − 0.2095x, and the determination coefficient (R^2^) was 0.8781 (Fig. 6A). The regression equation between the absolute content of gluconic acid and the seed germination was: y = 101.91 − 8.5837x, and the determination coefficient (R^2^) was 0.8748 (Fig. 6B). On this basis, we also determined a three-variable linear regression equation of seed germination (y) and galactose (× 1), gluconic acid level (× 2) and glycerol (× 3), which was y = 97.5831 − 0.1739 × 1 − 1.4513 × 2 − 2.2446 × 3, with a determination coefficient of 0.8966. The three-variable regression equation was considered to be more accurate than the single-variable equations.

## Discussion

### Seed Storability in Different Hybrid Combinations Might be Related to the Male Parent Restorer Line

The production of high-quality seeds is the primary aim of the seed industry; however, seed deterioration during storage is a considerable problem that increases crop production costs. Eliminating or minimizing losses from seed deterioration under storage is thus a priority of the seed industry. Seed vigor losses are typically believed to be related to harvesting and drying processes, together with the storage conditions (Nishizawa et al. 2008). Various engineering and technological approaches, such as low temperature and low humidity techniques, have been explored for maintaining seed integrity under long-term storage. However, low-temperature storage is costly and is not completely effective, and the safe storage of germplasm resources and food poses another challenge (Towill 2002). A study from the U.S. National Plant Germplasm System assessed the seed vigor of 42,000 samples stored in genebanks for 16–81 years and found that the average seed germination percentage decreased from the initial 91–58% (Wang et al. 2011; Chen et al. 2013).Improving seed storability and prolonging seed vigor have thus become increasingly relevant. Rice varieties vary in storability, and seed storability is affected by genetic factors as well as environmental factors. Employing a genetics approach provides a cost-effective means of addressing the decline in seed germination ability during storage.

A large number of studies have reported that the storage tolerance of inbreed rice seeds is better than that of hybrid rice seeds (Ellis 1993; Chen 1994; Gao et al. 2016). It is believed that the physiological advantage of easy germination in hybrid rice seeds is a primary factor contributing to poor storage tolerance (Chen 1994). Some experiments have also demonstrated that the storage tolerance of different hybrid combinations is closely related to their parents; for example, the restorer line Guanghui 122 was found to be associated with high storability (Liu et al. 2005)**.**

Our study showed a close association between the restorer lines and the storage tolerance of the tested combinations, which might provide a reference for the breeding of rice storage tolerance. For example, combinations from the high-storability restorer line Guanghui 122 demonstrated high storage resistance, with an average seed germination after two years of natural aging of 87%, which was significantly higher than the average value of the other restorers (Fig. 1). Guanghui 998 and Guanghui 368 have moderate storability, and their derived hybrid combinations showed weak storability (Fig. 1).

Notably, the storage capacities of the four cross combinations derived from Guanghui3618 differed significantly (Fig. 1). After storage, the germination percentage of TY-998 was 81%, indicating that its storage capacity was high, while that of IIY-3618 was the lowest at 40%, and those of BIIIY-3618 and QY-3618 were 52 and 50%, respectively, which were also poor. It is unclear if this is directly due to the effect of the restorer line on storability, or whether the interaction between the restorer and sterile lines affects the function of the storage tolerance genes. These questions are worthy of further study.

The screening of these long-life hybrid rice combinations has provided an excellent foundation for further exploring the associated storability genes and clarifying the mechanism of seed storage resistance, which is of great theoretical and practical significance to seed biology.

### Galactose and Gluconic Acid Constitute Reliable Metabolite Markers for Rice Seed Vigor and Aging

During the sale and transportation process of rice seeds, it is common for seeds to encounter high temperatures and humid conditions, causing the seeds to undergo inevitable and irrevocable changes in vigor, mean seed deterioration, or seed aging (Hampton and Tekrony 1995; McDonald 1999). The germination speed of aged seeds is slow, and the resulting seedlings will typically be non-uniform or will fail to emerge in stressful environments (Demir et al. 2008; Khajeh-Hosseini et al. 2009; Mavi et al. 2010). The rapid identification of seeds suitable for long-term storage or that need to go to the market immediately would help improve the decision-making process of the industry and avoid losses due to storage.

Several biochemical tests have been used as indicators of seed quality, including the redox indicator tetrazolium (Hampton and Tekrony 1995), enzyme activity assays(Ramiro 1995), and the determination of volatile compounds(Zhang and Roos 1997). Metabolites have great potential use as biomarkers, as it is relatively easy to develop diagnostic tests for their detection. A recent study showed that the galactinol content of mature dry seeds can be used as a biomarker for seed longevity in Brassicaceae and tomato (de Souza Vidigal et al. 2016). However, our study found no relationship between galactinol level and rice seed storability and/or vigor. We discovered that the relative raffinose level in the seeds before aging was significantly positively correlated with seed germination after artificial aging. As seed germination after artificial aging is the index used to measure seed vigor, the relative content of raffinose in the unaged seeds could be used to evaluate the vigor among different varieties. This result confirms previous conclusions (Yan et al. 2018), but differs from others (Bentsink et al. 2000).The underlying mechanism needs further study.

In the present study, the relative contents of galactose, fructose, gluconic acid, and glycerol in all the samples increased significantly following natural aging for 24 months (Fig. 4A–D). Among these, galactose and gluconic acid exhibited the most significant changes between the aged and unaged seeds. Furthermore, the absolute content of galactose and gluconic acid was negatively correlated with seed germination percentage, and low-vigor seeds showed high galactose and gluconic acid content, thus they could be used as candidate markers for seed aging.

We used regression equations to predict the seed germination corresponding to different metabolite contents (Fig. 6). In comparison with the conventional germination test, this method greatly saved time, used fewer seeds, and improved the testing efficiency. Future studies should improve the regression curves based on the seed characteristics of different combinations or varieties to increase the reliability of rapid testing.

Notably, the correlation between galactose and gluconic acid was strong (0.984), with both metabolites exhibiting nearly the same trend. Galactose is an important intermediate substance for ascorbic acid synthesis, while gluconic acid has anti-aging effects (Katagata 2011; Wang et al. 2013), which might explain their association with seed aging. A more interesting speculation is that galactose and gluconic acid form lactobionic acid (4-O-β-galactopyranosyl-d-gluconicacid), and lactobionic acid can prevent oxidative damage to the cell membrane. Lactobionic acid has roles in anti-aging, anti-oxidation, and promoting metabolism (Bai et al. 2012), which may provide insight into the subsequent clarification of related mechanisms.

## Conclusion

Seeds of different hybrid rice combinations significant differences in deterioration during natural and artificial aging. The combinations derived from Guanghui 122, a storage-resistant restorer, showed the highest germination percentage under both natural and artificial storage. A total of 89 metabolites and 56 metabolites were identified in the storage-tolerant and storage-intolerant hybrid rice combinations, respectively, most of which were related to primary metabolism. During the aging process, the content of galactose, fructose, glycerol, gluconic acid and other substances increased significantly in both high vigor and low vigor hybrid combinations. Absolute quantification indicated that galactose and gluconic acid were highly significantly negatively correlated with the germination percentage of the seeds under the different aging treatments, which suggested that these metabolites could constitute potential metabolic markers of seed vigor and aging. These findings are of great significance for the rapid and accurate evaluation of seed aging, the determination of seed quality, and the development of molecular breeding approaches for high-vigor rice seeds.

## Methods

### Plant Materials

The restorer lines Guanghui 122, Guanghui 998, Guanghui 3618, and Guanghui 368, and the sterile lines Qiu A and Tianfeng A were obtained from Guangdong Golden Rice Seed Industry Co. Ltd (Guangdong, China), and the sterile lines II-32A and BIIIA were obtained from the Guangxi Academy of Agricultural Sciences (Guangxi, China). The four restorer lines were combined with the four sterile lines to generate 16 hybrid rice combinations (Additional file 2: Table S1). Other materials including Guang 8 You 165 (G8Y165), NanxiuRuanzhan (NXRZ), NanYouzhan (NYZ), Guang 8 B (G8B), Huazhan (HZ), R Xiangzhan (RXZ), R534, NongLiangYou Huazhan (M2YHZ), YLiangYou 1 (Y2Y1), JifengYou 1002 (JFY1002) used for absolute quantification were obtained from Golden Rice Seeds Co. Ltd. of Guangdong, Yuan Longping High-tech Agriculture Co. Ltd. and Huamao Agricultural Development Co. Ltd.

### Artificial Aging Treatment

The artificial aging approach was based on the previous methods with slight modifications (Jun et al. 2000; Chen et al. 2012). The harvested rice seeds were divided into a control group and treatment group. The seeds in the treatment group were artificially aged as follows: the rice seeds were pre-treated at 15 °C under 85% relative humidity (RH) for 3 d, and then transferred to an incubator at 43 °C under 85% RH for 8 d, following which they were dried at 25 °C under 32% RH for 3 d. The seeds of the control group did not undergo any aging treatment.

### Natural Aging Treatment

The natural aging treatment of the seeds followed the method of Gao et al. (2016). Briefly, the seeds were harvested, dried, and stored for 12 months or 24 months under natural conditions (below 28 °C and 65% humidity using an air conditioner). The germination percentages were then determined.

### Seed Germination Test

Fifteen grams of the dried seeds from each treatment were weighed and pre-soaked in water for 12 h. One-hundred seeds from each treatment were assessed for germination percentage. The seeds were placed in a germination dish with two layers of moist paper at the bottom. During germination, the dish was kept in a 15,000 lx light incubator (12 h light/12 h dark). The germination potential after 4 d and the germination rate after 7 d were calculated. The experiment included three biological repeats.

Combining the relevant international rules for seed testing for the determination of vigor (International Seed Testing Association 1999) with actual practice of seed market, we defined high vigor seeds as the seeds whose germination percentage was higher than 80% after natural and artificial aging. Low vigor seeds were defined as those with a germination percentage less than 60% after aging. Seeds with values in between were defined as moderately vigorous seeds.

### Metabolite Profiling by GC–MS

Metabolites were extracted from 50 mg of the hybrid rice seeds using the protocol described by our previous study (Yan et al. 2018). The 300-μL extract solutions were dried in a vacuum concentrator for GC–MS analysis. The dried extracts were derivatized with *N*-methyl-*N*-(trimethylsilyl) trifluoroacetamide (MSTFA) as described previously by Yan et al. (2018), and transferred to glass vials for GC–MS (7890A-5975C, Agilent Technologies, Santa Clara, CA, USA) analysis. One µL from each sample was injected into the GC–MS (7890A-5975C, Agilent) at 270 °C in split mode (50:1) with the helium carrier gas (> 99.999% purity) flow set to 1 mL/min. A DB-35 MS (30 m × 0.25 mm, 0.25 µm) capillary column was used for separation. The temperature was isothermal for 5 min at 85 °C, followed by an 8 °C per min ramp up to 205 °C, where it was held for 5 min, and then finally ramped up at 8 °C per min to 300 °C and then held for 5 min. The transfer line temperature was set to 280 °C, and the ion source temperature was set to 230 °C. The mass range analyzed was from *m/z* 60 to 1000.

### Qualitative Analysis of Sugar Metabolites in Rice Seeds

Sugar metabolites were extracted from 100 mg of hybrid rice seeds using the protocol described by Yan et al. (2018), and a 200-μL extract solution was dried in a vacuum concentrator for GC–MS analysis. The dried extract was derivatized with MSTFA, as described previously and then transferred into glass vials. One μL from each sample was injected into the GC–MS with the same parameters as described above. The temperature was isothermal for 4 min at 90 °C, followed by an 8 °C per min ramp up to 205 °C, where it was held for 2 min, and then finally ramped up at 15 °C per min to 310 °C and maintained for 5 min. The transfer line temperature was set to 300 °C, and the ion source temperature was set to 230 °C. The mass range analyzed was from *m/z* 85 to 700. The MS analysis was conducted in selected ion monitoring (SIM) mode, and the quantitative ions are listed in Additional file 3: Table S2.

### Data Analysis

Chroma TOF 4.3X software (LECO Corporation) was used for GC–MS data analyses including peak extraction, data baseline filtering, calibration of the baseline, peak alignment, deconvolution analysis, and comparison of the peak area. The LECO-FiehnRtx5 database and the NIST library were used for peak identification (Kind et al. 2009). First, missing values in the raw data were placed with 50%the minimum value, and then the detected peaks were retained using the interquartile range denoising method. In addition, an overall normalization method was employed in the data analysis. The resulting three-dimensional data involving the peak number, sample name, and normalized peak area were fed to the SIMCA software package (V14, Umetrics AB, Umea, Sweden) for multivariate statistical analyses, including principal component analysis (PCA), orthogonal partial least squares-discriminant analysis (OPLS-DA), and permutation analysis of different models. To refine this statistical analysis for significantly changed metabolites, the first principal component of the variable importance projection (VIP) was obtained. The metabolites with |p(corr)|≥ 0.5 and VIP > 0.5 were considered different between the two comparison groups (Yan et al. 2018).

Data are presented as the mean ± SE of three replicates. One-way analysis of variance was used to compare mean values, and individual means were compared with the Fisher’s least-significant difference test (*p* < 0.05).

## Supplementary Information

**Additional file 1 Fig. S1**. The score plots generated from the PCA model from GC-MS spectra data, demonstratingthe dynamic metabolome change for the sixteen hybrid rice cultivars before and after the 24-month storage period.

**Additional file 2 Table S1**. Hybrid rice combinations formed by crossing 4 sterile lines and 4 restorer lines.

**Additional file 3**.** Table S2**. The retention time, qualitative ions and quantitative ions of metabolites detected in SIM mode.

**Additional file 4**:** Table S3-6**.** Table S3**. Metabolites for discriminating between the BII-0M group and the BII-24M group based on the multivarivate analyse of the untargeted metabolomics data.** Table S4**. Metabolites for discriminating between the IIY-0M group and the IIY-24M group based on the multivarivate analyse of the untargeted metabolomics data.** Table S5**. Metabolites for discriminating between the QY-0M group and the QY-24M group based on the multivarivate analyse of the untargeted metabolomics data.** Table S6**. Metabolites for discriminating between the TY-0M group and the TY-24M group based on the multivarivate analyse of the untargeted metabolomics data.

**Additional file 5**.** Table S7**. Ratio of relative contents of various carbohydrate substances in rice seeds after/before storage.

**Additional file 6**.** Table S8**. Correlation coefficient between seed germination percentage and sugar content in seeds.

**Additional file 7**.** Table S9**. Absolute contents of 8 sugars in rice seeds under different natural aging degrees and their correlation with germination percentage.

## Data Availability

All data generated or analyzed in this study are included in this published article and its additional information files.
