# Supplementary material for: Identification of Metabolomic Biomarkers of Seed Vigor and Aging in Hybrid Rice
Source: Rice (N Y). 2022 Jan 27;15:7. doi: 10.1186/s12284-022-00552-w (PMC8795261; doi:10.1186/s12284-022-00552-w)

Fig. S1. The score plots generated from the PCA model from GC-MS spectra data, demonstrating the dynamic metabolome change for the sixteen hybrid rice cultivars before and after the 24-month storage period.


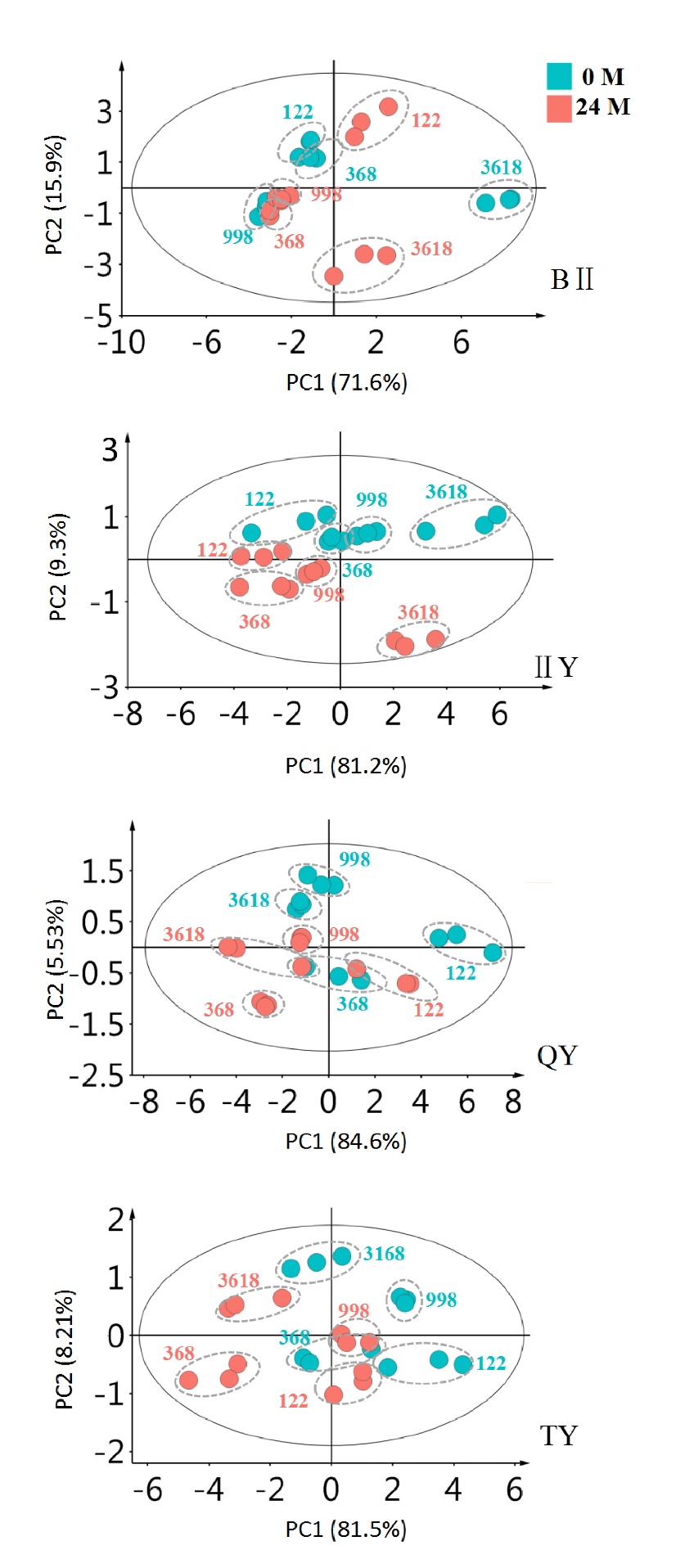

Supplement: Supplementary file 1 — Additional file 1 Fig. S1. The score plots generated from the PCA model from GC-MS spectra data, demonstratingthe dynamic metabolome change for the sixteen hybrid rice cultivars before and after the 24-month storage period. [file 12284_2022_552_MOESM1_ESM.docx]
