# Supplementary material for: Identification of Metabolomic Biomarkers of Seed Vigor and Aging in Hybrid Rice
Source: Rice (N Y). 2022 Jan 27;15:7. doi: 10.1186/s12284-022-00552-w (PMC8795261; doi:10.1186/s12284-022-00552-w)
Supplement: Supplementary file 2 — Additional file 2 Table S1. Hybrid rice combinations formed by crossing 4 sterile lines and 4 restorer lines. [file 12284_2022_552_MOESM2_ESM.docx]

Table S1 16 hybrid rice combinations formed by crossing 4 sterile lines and 4 restorer lines

**Hybridized combination**

**Hybridized combination**

| **Sterile**  **lines**  **Restorer lines** | **BⅢ A** | **Ⅱ-32A** | **Tianfeng A** | **Qiu A** |
| --- | --- | --- | --- | --- |
| **Guanghui 122** | BⅢY-122 | ⅡY-122 | TY-122 | QY-122 |
| **Guanghui 998** | BⅢY-998 | ⅡY-998 | TY-998 | QY-998 |
| **Guanghui 368** | BⅢY-368 | ⅡY-368 | TY-368 | QY-368 |
| **Guanghui 3618** | BⅢY-3618 | ⅡY-3618 | TY-3618 | QY-3618 |
