# Supplementary material for: Identification of Metabolomic Biomarkers of Seed Vigor and Aging in Hybrid Rice
Source: Rice (N Y). 2022 Jan 27;15:7. doi: 10.1186/s12284-022-00552-w (PMC8795261; doi:10.1186/s12284-022-00552-w)
Supplement: Supplementary file 3 — Additional file 3. Table S2. The retention time, qualitative ions and quantitative ions of metabolites detected in SIM mode. [file 12284_2022_552_MOESM3_ESM.docx]

Table S2 The retention time, qualitative ions and quantitative ions of metabolites detected in SIM mode

| Analyte | Retention time (min) | Qualitative ions (m/z) | Quantitative ions (m/z) |
| --- | --- | --- | --- |
| Fructose 1 | 16.99 | 307,217,147 | 103 |
| Fructose 2 | 17.17 | 307,217,147 | 103 |
| Galactose | 17.241 | 319,147,103 | 205 |
| Glucose 1 | 17.345 | 147,205,160 | 319 |
| Glucose 2 | 17.602 | 147,205,160 | 319 |
| Glucopyranoside | 18.012 | 133,147,217 | 204 |
| Galactinol | 26.851 | 191,217,147 | 204 |
| Gluconic acid | 18.449 | 147,292,205 | 333 |
| Raffinose | 28.594 | 217,204,191 | 361 |
| Glycerol | 7.869 | 147,103,117 | 205 |
| Sucrose | 24.961 | 217,147,437 | 361 |
