# Supplementary material for: Identification of Metabolomic Biomarkers of Seed Vigor and Aging in Hybrid Rice
Source: Rice (N Y). 2022 Jan 27;15:7. doi: 10.1186/s12284-022-00552-w (PMC8795261; doi:10.1186/s12284-022-00552-w)
Supplement: Supplementary file 4 — Additional file 4: Table S3-6. Table S3. Metabolites for discriminating between the BII-0M group and the BII-24M group based on the multivarivate analyse of the untargeted metabolomics data. Table S4. Metabolites for discriminating between the IIY-0M group and the IIY-24M group based on the multivarivate analyse of the untargeted metabolomics data. Table S5. Metabolites for discriminating between the QY-0M group and the QY-24M group based on the multivarivate analyse of the untargeted metabolomics data. Table S6. Metabolites for discriminating between the TY-0M group and the TY-24M group based on the multivarivate analyse of the untargeted metabolomics data. [file 12284_2022_552_MOESM4_ESM.docx]

Table S3 Metabolites for discriminating between the BⅡ-0M group and the BⅡ-24M group based on the multivarivate analyse of the untargeted metabolomics data.

| **No** | **Compounds** | **P(corr)** | **VIP** |  |  |
| --- | --- | --- | --- | --- | --- |
| 1 | Glycerol | 0.970358 | 3.23241 | ↑ | BⅡ-0M < BⅡ-24M |
| 2 | Ethanolamine | 0.735473 | 0.614613 | ↑ | BⅡ-0M < BⅡ-24M |
| 3 | Galactose | 0.7291 | 3.64819 | ↑ | BⅡ-0M < BⅡ-24M |
| 4 | Gluconic acid 2 | 0.713818 | 1.58953 | ↑ | BⅡ-0M < BⅡ-24M |
| 5 | Gluconic acid 1 | 0.690197 | 1.64982 | ↑ | BⅡ-0M < BⅡ-24M |
| 6 | Phosphate | 0.615497 | 0.804233 | ↑ | BⅡ-0M < BⅡ-24M |
| 7 | Fructose 1 | 0.60896 | 2.83818 | ↑ | BⅡ-0M < BⅡ-24M |
| 8 | Fructose 2 | 0.574141 | 2.08499 | ↑ | BⅡ-0M < BⅡ-24M |
| 9 | Mannopyranose | 0.527037 | 0.591863 | ↑ | BⅡ-0M < BⅡ-24M |
| 10 | Glucopyranoside | -0.68734 | 1.86447 | ↓ | BⅡ-0M > BⅡ-24M |
| 11 | 2,4,6-Trifluoroaniline | -0.84441 | 0.700016 | ↓ | BⅡ-0M > BⅡ-24M |
| 12 | Oxalacetic acid | -0.875702 | 1.40539 | ↓ | BⅡ-0M > BⅡ-24M |

Table S4 Metabolites for discriminating between the ⅡY-0M group and the ⅡY-24M group based on the multivarivate analyse of the untargeted metabolomics data.

| **No** | **Compounds** | **P(corr)** | **VIP** |  |  |
| --- | --- | --- | --- | --- | --- |
| 1 | Ethanolamine | 0.873314 | 0.782975 | ↑ | ⅡY-0M <ⅡY-24M |
| 2 | Glycerol | 0.862942 | 3.15774 | ↑ | ⅡY-0M <ⅡY-24M |
| 3 | Gluconic acid 2 | 0.759262 | 1.46346 | ↑ | ⅡY-0M <ⅡY-24M |
| 4 | Phosphate | 0.749981 | 0.870956 | ↑ | ⅡY-0M <ⅡY-24M |
| 5 | Gluconic acid 1 | 0.715665 | 1.37875 | ↑ | ⅡY-0M <ⅡY-24M |
| 6 | Galactose | 0.708883 | 3.18362 | ↑ | ⅡY-0M <ⅡY-24M |
| 7 | Fructose 1 | 0.582857 | 2.31426 | ↑ | ⅡY-0M <ⅡY-24M |
| 8 | Myo-Inositol | 0.577864 | 0.803232 | ↑ | ⅡY-0M <ⅡY-24M |
| 9 | Mannopyranose | 0.564184 | 0.692762 | ↑ | ⅡY-0M <ⅡY-24M |
| 10 | Fructose 2 | 0.557949 | 1.76542 | ↑ | ⅡY-0M <ⅡY-24M |
| 11 | Talofuranose | -0.544911 | 0.512948 | ↓ | ⅡY-0M >ⅡY-24M |
| 12 | Glucopyranoside | -0.684786 | 1.96323 | ↓ | ⅡY-0M >ⅡY-24M |
| 13 | Oxalacetic acid | -0.888201 | 1.5469 | ↓ | ⅡY-0M >ⅡY-24M |

Table S5 Metabolites for discriminating between the QY-0M group and the QY-24M group based on the multivarivate analyse of the untargeted metabolomics data.

| **No** | **Compounds** | **P(corr)** | **VIP** |  |  |
| --- | --- | --- | --- | --- | --- |
| 1 | Glycerol | 0.980273 | 2.38873 | ↑ | QY-0M <QY-24M |
| 2 | Gluconic acid 2 | 0.956765 | 0.96951 | ↑ | QY-0M <QY-24M |
| 3 | Ethanolamine | 0.937405 | 0.566498 | ↑ | QY-0M <QY-24M |
| 4 | Gluconic acid 1 | 0.935601 | 1.09 | ↑ | QY-0M <QY-24M |
| 5 | Galactose | 0.930138 | 2.15571 | ↑ | QY-0M <QY-24M |
| 6 | Phosphate | 0.741019 | 0.552325 | ↑ | QY-0M <QY-24M |
| 7 | Fructose 1 | 0.702992 | 1.39887 | ↑ | QY-0M <QY-24M |
| 8 | Hydroxylamine | 0.65474 | 1.02763 | ↑ | QY-0M <QY-24M |
| 9 | Fructose 2 | 0.612661 | 0.899143 | ↑ | QY-0M <QY-24M |
| 10 | Glucopyranoside | -0.622953 | 1.07853 | ↓ | QY-0M >QY-24M |
| 11 | Oxalacetic acid | -0.730551 | 0.91213 | ↓ | QY-0M >QY-24M |

Table S6 Metabolites for discriminating between the TY-0M group and the TY-24M group based on the multivarivate analyse of the untargeted metabolomics data.

| **No** | **Compound** | **p(corr)** | **VIP** |  |  |
| --- | --- | --- | --- | --- | --- |
| 1 | Gluconic acid 2 | 0.978953 | 0.681028 | ↑ | TY-0M <TY-24M |
| 2 | Glycerol | 0.970823 | 2.17586 | ↑ | TY-0M <TY-24M |
| 3 | Galactose | 0.966984 | 1.45191 | ↑ | TY-0M <TY-24M |
| 4 | Gluconic acid 1 | 0.843744 | 0.673751 | ↑ | TY-0M <TY-24M |
| 5 | Fructose 1 | 0.520253 | 0.631521 | ↑ | TY-0M <TY-24M |
| 6 | Sucrose | -0.568508 | 8.35271 | ↓ | TY-0M >TY-24M |
| 7 | Maltose 2 | -0.639033 | 0.747236 | ↓ | TY-0M >TY-24M |
| 8 | Maltose 1 | -0.654933 | 1.44378 | ↓ | TY-0M >TY-24M |
| 9 | Oxalacetic acid | -0.733152 | 0.741321 | ↓ | TY-0M >TY-24M |
| 10 | Glucopyranoside | -0.825393 | 0.682249 | ↓ | TY-0M >TY-24M |
